# Exploring the relationship between housing concerns, mental health and wellbeing: a qualitative study of social housing tenants

**DOI:** 10.1093/pubmed/fdz076

**Published:** 2019-07-11

**Authors:** Eleanor Holding, Lindsay Blank, Mary Crowder, Edward Ferrari, Elizabeth Goyder

**Affiliations:** 1 The School of Health and Related Research (ScHARR), The University of Sheffield, Regent Court, Regent Street, Sheffield, UK; 2 Centre for Regional Economic Social Research (CRESR), Sheffield Hallam University, 54 Howard St, Sheffield, UK

**Keywords:** housing, social housing, mental health

## Abstract

**Background:**

The rising prevalence of mental health problems is a growing public health issue. Poor mental health is not equally distributed across social groups and is associated with poverty and insecure housing. An evaluation of a social housing intervention provided an opportunity to explore the connections between housing and wider determinants of health and wellbeing.

**Methods:**

We undertook 44 interviews with social housing tenants over a two-year period to explore their views on housing, health and wellbeing.

**Results:**

Poor mental health was common. The results suggest that perceptions of housing quality, service responsiveness, community safety, benefit changes and low income all have a detrimental effect on tenants’ mental health.

**Conclusions:**

Social housing providers who wish to have a positive impact on the mental health of their tenants need to consider how to best support or mitigate the impact of these stresses. Addressing traditional housing officer functions such as reporting or monitoring home repairs alongside holistic support remains an important area where social housing departments can have substantial health impact. Tackling the complex nature of mental health requires a joined up approach between housing and a number of services.

## Introduction

The rising prevalence of mental health problems is a growing public health issue^1^. A 2014 survey of mental health and wellbeing in England found that 1 in 6 people over the age of 16 had experienced symptoms of a mental health problem, such as depression or anxiety, in the previous week^2^. Although there has been a general population increase in prevalence of poor mental health it is not equally distributed across social groups^3^ and is associated with lower socioeconomic status and poverty^4^. Progress in reducing poverty in the UK has stalled, with 22% of the population living in poverty in 2015/16, rising from 20% in 2004^4^. Poverty is also closely associated with living in poor quality or insecure housing^5^, another risk factor for poor mental health.

**Table 1:** Characteristics of sample (*n* = 39, rounded percentages).

Sample characteristics	*N*	%
**Gender**		
Male	16	41
Female	23	59
**Age group (years):**		
20–29	4	10
30–39	6	15
40–49	4	10
50–59	8	21
60–69	6	15
70–79	7	18
80 and over	2	5
Not known—participant would prefer not to say	2	5
**Employment status**		
Employed	7	18
Unemployed	14	36
Retired	7	18
In higher education	1	3
Full time carer	3	8
Off work due to sickness and/or disability	7	18
**Living arrangements**		
Lives alone	18	46
Lives with partner (no children)	8	21
Lives with partner and children	4	10
Lives with children	6	15
Lives with others	3	7

Those living in social housing are particularly vulnerable. A recent review found that 45% of social housing tenants in England were earning the lowest fifth of income, with 44% living in poverty after housing costs were taken into consideration^6^. 37% of social renters were in employment compared to 59% of the population whilst 30% were retired and 23% were off work due to sickness, being in education or caring responsibilities compared to 29% and 9% of the overall population^6^. In addition, those in social housing are 1.5 more times likely to suffer with poor mental health^7^, and four times more likely to report that housing conditions worsen their health^8^. Although the makeup of social renters is broadly similar across Europe, tenancies in England tend to be a less secure and stable type of tenure due to probationary tenancies—‘trial’ tenancies where social renters can be easily removed if they do not adhere to the terms and conditions.^9^

Despite the recognized link between housing and mental health, local government’s ability to exert change is limited by the continued austerity agenda, budget constraints and policy decisions made at a national level. Housing and homelessness remain second in terms of council budget priorities after adult social care, but *‘austerity has pushed council budgets to breaking point*’ and budgets are set to further reduce according to the latest Local Government Finance Survey.^10^ The rising prevalence of mental health conditions alongside increased cuts to public spending requires a joined up, preventative approach across a number of services.^11^

This study is based in a large Northern city where the Council is the largest provider of social rented homes, owning and managing 40 195 properties (16.5%). In 2017 the Council rolled out its ‘Housing+’ programme after an initial pilot in the south east of the city. The new service allocates a dedicated housing officer to each tenancy and involves undertaking an annual home visit with a geographically based caseload of between 180–330 households. Visits are designed to discuss wider determinants of tenancy sustainability such as health, crime, community engagement, employment and finances. The driver behind this change was to provide a more efficient service whilst also recognizing the varying and often complex support needs of social housing tenants.

Interest in evaluating the impact of the Housing+ service presented us with an opportunity to explore the connections between housing, mental health and wellbeing for social housing tenants. Although there is no consensus on a single definition of wellbeing there is general agreement that it includes the presence of positive emotions, life satisfaction and positive functioning.^12^ This study presents the views of 39 social housing tenants receiving the new service on housing conditions, health and wellbeing, framed within a wider two-year evaluation of the Housing+ programme. Although this study focuses on a particular initiative and geographical area, the issues raised are far from unique—and will apply to many social housing estates and providers across the country.

## Methods

### Sample and data collection

Qualitative interview participants were recruited via a telephone survey administered by a commercial research company. Participants for the telephone survey were selected in order to provide a broad representation of the tenant population in terms of age, ethnicity and property type. The telephone survey asked a series of questions on health and wellbeing, and sought consent to provide contact details to the research team. Contact details of consenting participants were sent securely to the research team weekly during the recruitment period (Oct-Dec 2016). All respondents agreeing to further participation were then contacted to invite them to participate in a face-to-face interview at a time and place convenient for them. As such, the sample selection was purposive for the telephone survey, with a convenience sub sample being selected for interview.

31 semi-structured interviews were conducted as part of Year 1 of the evaluation, using a topic guide covering demographics, their current situations in terms of health, employment and other factors, and their views and experiences of the Housing+ programme. A further 13 interviews were undertaken in the second year of the evaluation (5 with repeat participants/8 new). Recruitment of new interviewees took place between October and December 2017 using the same methods as the previous year. All interviews lasted between 30 minutes and an hour and a half (mean duration: 45 minutes). Interviews were conducted until discourse saturation.

## Analysis

With the participants’ informed consent, the interviews were digitally recorded. Interviews were transcribed verbatim and anonymised before coding in NVivo 10 software. An initial coding framework was derived by the research team from the in-depth reading of a small number of transcripts, subsequently modified to reflect the emerging themes. The researchers met regularly to discuss the suitability and validity of the coding framework, including any disagreements, before the coding framework was finalized. The data were analysed using framework analysis to organize the data into key themes according to the policy interests of the Local Authority.^13^ A theory of change model was employed in this analysis to explain how the intervention is expected to bring about the desired results rather than just describing the results.^14^

## Results

### Participant characteristics

The mean age of the tenants interviewed was 47 years (range 22 to 96). 23 out of 39 participants were female (59%). The composition of tenants’ households varied but just under half of the sample (18 tenants, 46%) lived alone. The remainder comprised a mix of couples with children, single parent families and multiple adult households. Tenants had lived in their current properties between 7 months and 72 years, with the majority living in the same property for over 10 years. Most had housing histories that involved moving in and out of council housing for most of their lives (Table 1).

### Health issues and health service use

Almost all tenants discussed personal or household health and wellbeing issues, with many describing their health as ‘*poor*,’ or ‘*up and down*’. Many participants and their families discussed mental health issues, alongside physical health problems. These included five tenants who reported suffering from depression, of which three took medication, one received counselling and one is supported by local mental health services. Other mental health conditions experienced by participants were anxiety, schizophrenia and agoraphobia. One participant, who was receiving support from mental health services, told of having been previously hospitalized due to paranoid schizophrenia. Participants also made more general comments on how they were *‘struggling*’ with their mental health and wellbeing or described depressive symptoms at interview, although they had not received a formal diagnosis. For instance, one participant described how they often *‘feel absolutely crap*’ and either want to do *‘nothing*’ or *‘go to [the] boozer and get drunk*.’

### Housing conditions and repairs

Most interviewees had either spoken to their housing officer about issues with the condition of their housing and more specifically relating to needed repairs, or otherwise said that they would want to discuss them if they received a visit. Issues identified by participants ranged from loose plasterwork, leaking windows, baths, toilets, showers and water pipes, cracked roofs, broken doors, uneven floorboards and damp, cold and overcrowded housing.

Interviewees gave specific examples of delays to the repairs service, with long periods of time—sometimes years before the completion of jobs. Such delays caused what participants described as *‘frustration*’, *‘stress*’ and *‘upset*’, with one participant saying they have now given up reporting repairs as it *‘wastes your time and wastes your energy and things don’t get done’.* In the majority of cases participants put emphasis on the responsibility of the council repair service to deal with housing and repairs issues. Due to continued delays one family had used their own resources to deal with repairs incurring further *‘stress*’. A couple of tenants noted that damp within their property was having a negative impact on their physical and mental health, both in terms of aggravating existing physical health problems and causing stress and worry (direct quotes given in Box 1).

Box 1 Housing conditions and repairs

**Table:** 

*I never, ever have colds and bad chests and things like that. I have got illnesses but never related to my chest. But the damp in here, which I can show you pictures of, you know, are horrific…I think, I don’t know whether I had three inspectors or four inspectors that came to look at it. And everyone said, yes, we’ll get it dealt with straightaway. Well, straightaway is six months down the line and you’re still living with this damp.’ (Female, 65, unemployed)* *‘Over that three years we were living here we didn’t have central heating upstairs at all. My little one who was just, like, one year old at that time, I complained so many times to the council. I went to the doctors; I had support letters, nobody… some people was coming to check. They never, you know, like, put it in the system. Whenever they come here they don’t put in. So, I was complaining, complaining, complaining. Two weeks ago they’ve just been putting it off’ (Female, 34, stay at home single mother)* *‘The water came through the lighting and through the actual… and it just flooded the downstairs toilet. So they then came out and said it’s just fine, it will dry on its own. And then we had the like just intermittent repairs…and we had this problem with this guy and we said we want a new plumber to come out. So then we had an inspector come out just recently and said oh we’re going to have to put it back on a 55 day job even though there had been ongoing problems knowing that we needed this bath and shower for our child who’s got a disability…but they said bath wasn’t a priority, they told us it wasn’t a priority whatsoever. So I was really getting quite upset about it’ (Female, 30, full time carer)* *‘Because I know my priorities in the house. I pay rent. I make sure the house is clean. Anything to do with repairs is up to them, to the council. It takes a while, you know, to argue well this and that, and you might get it done, sometimes it might take two or three years to get it done… sometimes somebody will be sent and they will be doing the different things for the same place and you think that wasn’t what I phoned to be done’ (Female, 26, stay at home mother)*

### The wider built environment: neighbourhood conditions, safety and mental health

Crime and antisocial behaviour on housing estates, including drug dealing and gang fights, were mentioned by some participants, resulting in stress and reduced feelings of safety and belonging. Two participants who were both young described their desire to move from the area due to safety concerns for their children. Another participant was scared to leave her house due to the antisocial and violent behaviour of her neighbours towards herself and her daughter.

Some participants discussed the wider conditions of the neighbourhood and its effect on feelings of stress. Littering and rubbish were issues for some participants, with one participant describing how neighbours would continuously throw bottles, cans, food packets and cigarettes over the wall into her front garden. One interviewee reported that despite the council street cleaning twice a week they were aware of resultant rodent infestations and believed that these circumstances were linked to local businesses shutting down or leaving the area (direct quotes given in Box 2).

Box 2 The wider built environment: neighbourhood conditions, services, safety and mental health

**Table:** 

*‘But the safety of the area it’s not, I’m not happy with it… I’ve been thinking to move away from it the last couple of years to be honest. But now because I’ve got children…well really, really I need to…Mainly there’s a lot of drug dealers, gun fire, just like this, outside my window. Somebody’s been killed. The other day I could see them fighting with the guns… And sometimes you come and you will find people on the stairs with the needles and there’s blood on the floor. They will be sick, they will be pooing if they want to.’ (Female, 26, stay at home mother)* *‘They were bullying my daughter. We used to walk down the road and the neighbours were, like, all friends and they had parking issues. We’d like to park in front of our house but people got there first and they were just arguing. We had the police… One of the neighbours wrote a letter saying my daughter is going to die. We phoned the police and they did nothing…The council asked the police if the neighbours were a threat and the police said: no. We wanted to move and everything because it wasn’t very nice’ (Female, 46, off work due to sickness and disability)* *‘To be honest the cleanliness of the place is because we live [unclear] local take always, it causes a really really big problem. The council cleans twice a week…. We did have a big problem with a mouse, a rat, and a few shops have been shut down as well, close to us.’ (Female, 26, stay at home mother)* *‘I’m fed up. Every day I have to go out there and pick lager cans up, cigarette packets, they’ll be thrown over into my garden. I don’t even go up there; I daren’t think what’s thrown off there. And [the council] say we can’t help’ (Female, 65, unemployed)* *‘No, to be honest because there’s nothing here the children could… even the park, we’ve got a little park in here behind the church and it’s empty. We don’t have swings, we don’t have slides. When the children are bored in the house and they fight most of the time the kids fight because of TV. I try to take them out because fresh air and play. But there’s nothing for them to play in our areas.’ (Female, 34, stay at home mother)*

### Income, benefit cuts and rent arrears

Several tenants discussed their employment and financial situations. Eighteen percent were working either part-time or full-time, but the majority of the participants were not in current employment and claimed benefits for themselves or on behalf of their family members. Several participants discussed their financial struggles, describing their situations as *‘just surviving*’ or *‘just coping*’, with five participants stating they had been in mild or severe rent arrears, with one receiving a court order because of this. Two participants, both classified as disabled due to both physical and mental health issues, had encountered difficulty claiming their Personal Independence Payments (PIP) due to strict medical assessments which they had to attend every 18 months.

Some participants also described the relationship between income and other factors such as food security and reliance on food banks. For example, one participant also described how her financial situation prevented her from leaving her area as she was unable to afford transport costs, contributing to her feelings of isolation and loneliness (direct quotes given in Box 3).

Box 3 Income, benefit cuts and rent arrears

**Table:** 

*‘I mean, you should pay rent because it’s roof over your head. I understand that. And in times of trouble, my dad’s always said, if you speak to people, you get it sorted quicker and the stress is off your head. But when you’re in the turmoil, it’s a different kettle of fish. Because you’re like, well I don’t know what way to do it…If I pay that, I’ve got nothing left. Then I have to go and borrow. But the time the next payment comes round, you’re skint again. And my mum and dad are a pensioner now, so they can’t afford. So you’re like, well what do I do? I had a food package delivered…I think I’ve had that twice’ (Female, 34, unemployed)* *‘I’ve left [my job]… my family don’t want me there. They say I look better and things, but they don’t know how soul destroyed I am… It’s. I struggle mentally. I came close to having a breakdown. Up to last Sunday I hadn’t had a day without a drink this year… But it got to the point where it was at least a bottle a night, every single night…I think it’s stress, life, everything.’ (Female, 57, employed)* *‘Assessments. They sit you in these assessments, badger-speaking to you. Little few exercises that are barely motivating at all. And they throw you off, there’s nothing wrong with you. So, all of a sudden, after 20-some years, all my osteoarthritis and arthritis in my spine, and arthritis in my fingers and my feet, has disappeared. I’m not disabled anymore, and I’m fit for everything…[it’s] very hard. I’m having to use food banks which, they told me last week at Citizen’s Advice, because I’ve had three vouchers and more, I’m not entitled to any more now. So, when I went to the food bank, I said, I asked [a worker there]… She said, why would they say that? What happens when you’ve really got nothing? I said, well, it’s like that now…Tinned stuff. Beans, tomatoes, eggs. That’s what I can buy, or what people give me.’ (Female, 45, off work due to sickness and disability)*

## Discussion

### Main findings of this study

In the course of an evaluation of a social housing intervention in a major UK city, what emerged was a strong focus on the relationship between housing maintenance, the wider built environment, income and benefit changes and the mental health of tenants. This suggests that the mental health and wellbeing of social housing tenants is influenced by a wide range of complex, structural and psychosocial processes.

Our research highlights the complex relationships between, and the impact on tenant health from a number of interrelated factors. These primarily include the affordability of and satisfaction with living conditions; the physical condition and state of repair of the home; the physical environment; and the social environment of the neighbourhood. In order to improve tenant wellbeing in the long term, all of these issues must be tackled together.

### What is already known about the topic

Mental health is a significant public health problem and is closely associated with poor quality housing.^5^ Previous research has identified causal links between housing, the built environment and aspects of mental health and wellbeing (e.g.^15,16,17,18^). Polluted environments and dark public spaces have been linked to aggressive behaviour^17^ while high rise living, crime, local amenities, area satisfaction and reputation contribute to anxiety and depression.^15,19,20^ In contrast, adequate income and positive perceptions of neighbourhood safety are linked to positive mental health outcomes.^21^ Although previous research has investigated the link between housing conditions and mental health, qualitative approaches remain underdeveloped.

Individuals across different forms of housing tenure face a variety of problems that increase the risk of poor mental health.^22^ A study of the impact of tenure type on mental health identified a range of factors that can impact differentially across social housing, the private rented sector and home ownership.^23^ Like social housing tenants, homeowners and private renters may also have little control over property repairs due to dependency on landlords or lack of household income.

### What this study adds

Despite similarities between the social and private rented sector, social housing providers may be better placed to understand and mitigate housing related stresses through initiatives such as Housing+. What this study adds is an understanding of how social housing providers offering interventions such as Housing+ may improve the mental health and wellbeing of their tenants.

Increased attention is being given to the potential of housing services to provide preventative support to reduce demand on health and social care services.^24^ Housing+ is one such approach aiming to provide holistic support for tenants where the focus is on early intervention and dealing with low level issues directly in order to prevent tenants reaching crisis point. Although the composition of ‘Housing Plus’ activities varies by organization, similar preventative strategies are being employed by housing services nationally. Through regular contact and home visits housing staff provide early intervention in areas that negatively impact mental health. For example, financial support may be offered before a tenant reaches rent arrears or housing quality is improved through the prioritization of early stage repairs.

Our results indicate that providing decent homes and timely repairs alongside support to address wider determinants of wellbeing is important for improved tenant mental health. Given these complex and interrelated factors social housing providers should consider how to connect with external agencies such as mental health support, financial services, the police and social services to provide preventative support for tenants. It is also clear that addressing traditional housing officer functions such as reporting or monitoring home repairs remains an important area where social housing departments can have substantial health impact, despite increased impetus to provide holistic support in other areas. Although home visits are designed to address wider determinants of wellbeing many tenants still wished to address issues consistent with traditional housing officer responsibilities. This was often prioritized over wider wellbeing issues which were often seen by tenants as outside of the responsibilities of the housing department.^25^ This demonstrates that social housing providers wishing to tackle mental health through holistic support should do so without losing focus on traditional functions. This is reliant on good internal communication and joint working between officers and the repair service. Carefully defining who is responsible for repairs and maintenance is also crucial to ensure tenant expectations and satisfaction are managed.

Our results highlight the importance of the wider built environment for the mental health of social housing tenants. Alongside discussing the housing conditions of their individual dwellings, tenants described how the safety, cleanliness, amenities and social environment of the wider estate impacted on their wellbeing. This indicates that the provision of high quality public or private housing does not guarantee positive health outcomes in the absence of adequate ecosystem services provided to residents.^26^ It also reinforces that neighbourhoods affect individual and community mental health through many interrelated pathways, including close proximity to essential amenities, services and green space, community support networks and social cohesion, and perceptions of housing quality.^27^

### Limitations of this study

Tenant participants were recruited via a telephone survey by a commercial research company as part of a wider evaluation of the Housing+ programme. This will have excluded those without a stable home, without a telephone and who were not available for interview due to work or other commitments. This meant that there is an over presentation of older and unemployed interviewees compared to the characteristics of social housing tenants as a whole.^5^ Our results are therefore based on a specific group of the population and should be interpreted with caution in terms of their generalizability. However, our sample is broadly representative of social housing tenants in terms of sex and those in single person households.^5^ Results from the baseline telephone survey also showed that the vast majority of respondents were white British (88%). However, sustained effort was made by the research team to recruit younger and BME participants by targeting specific geographical areas and conducting interviews in the evening to fit in with working schedules.

A systematic review of the literature linking housing improvements to improved health found evidence in this field to be relatively poor due to the difficulty of controlling for material deprivation.^16^ Similarly, although the results of this study indicate the importance of inadequate housing repairs for mental health it is difficult to assert cause and effect due to the interaction of deprivation with housing conditions and the wider conditions of the estate. Despite these limitations, this research draws attention to the relationship between housing and mental health through the views of social housing tenants themselves.

## Conclusion

Given the complexity of issues faced by social housing tenants alongside increased cuts to public spending, this paper considers the extent to which social housing providers are able to positively impact the mental health of their tenants. Housing conditions, the wider built environment, income and benefit changes impact on the mental health of social housing tenants. Social housing providers may be well placed to mitigate housing related stresses through joint working with external agencies to provide interventions aimed at empowering tenants to have more control in these areas. Providing decent homes and timely repairs still remains an important area where social housing providers can have a positive health impact, alongside support designed to address wider determinants of wellbeing.

While this qualitative study provides greater understanding of the interrelated processes that may influence the mental health of tenants, further mixed methods research is advocated to disentangle the complex relationship between housing and mental health.
